# Can External Neuromodulation Garments Improve Gait and Function in Children With Cerebral Palsy? A Prospective Single‐Arm Study

**DOI:** 10.1002/hsr2.70566

**Published:** 2025-03-23

**Authors:** Lindsey Jean Ross Weller, Shelly‐Anne Marie Sherwood, Shin Huey Ng, Maheswari Vellaichamy, Asila Alia Noordin, Ling Ying Tan, Arjandas Mahadev, Tong Hong Yeo, Zhi Min Ng

**Affiliations:** ^1^ Physiotherapy Department KKH Women's and Children's Hospital Singapore Singapore; ^2^ Division of Nursing KKH Women's and Children's Hospital Singapore Singapore; ^3^ Paediatric Orthopaedic Surgery KKH Women's and Children's Hospital Singapore Singapore; ^4^ Neurology Service, Paediatric Medicine KKH Women's and Children's Hospital Singapore Singapore

**Keywords:** cerebral palsy, electrical stimulation, spasticity

## Abstract

**Introduction:**

The Exopulse Mollii Suit is an external electrical stimulation garment that is designed to reduce spasticity through electrical stimulation of targeted muscles. Our aims were to study the impact of the garment in improving gait and function in children with cerebral palsy (CP).

**Methods:**

Individuals aged 4–18 years with spastic CP, Gross Motor Function Classification System level I–III were included for a prospective single‐arm study from January 2021 to January 2022. Participants wore the suit for 4 weeks 60 min a day. Outcome measures taken pre, post and 1‐month‐post intervention included: 3D gait analysis (gait profile score, gait deviation index and temporo‐spatial parameters), gross motor function measure‐88 (GMFM‐88), EQ‐5D‐Y, compliance rate, adverse event and satisfaction. Paired *t*‐test was used for data analysis to compare measurement time points.

**Results:**

Twenty children (median age 7 [range: 4–16; interquartile range: 3.1] years, 55% female, 45% male were recruited. Post‐intervention results showed there was no improvement in the gait profile but there was an improving trend in temporo‐spatial parameters GMFM Domain C *crawling and kneeling* improved significantly (*p* = 0.03). Improvement in EQ‐5D‐ Y *usual activity* was significant (*p* = 0.04). Compliance rate was 95% and nil major adverse event was reported. The majority (75%, *n* = 15) of parents and participants perceived overall positive experience.

**Conclusion:**

The positive changes in gait profile and function were no longer significant at 1‐month post‐intervention. Further studies with a longer intervention period and concurrent strengthening program are required.

**Implications for Physiotherapy Practice:**

Using the Molli Suit for 60 min a day for 4 weeks, may be useful in improving:(1) Gait cadence in children with CP. (2) Gross motor function in terms of crawling and kneeling in children with CP.

## 1 Introduction

Cerebral palsy (CP) is a neurological condition caused by nonprogressive brain injury occurring before, during or immediately after birth and up to 2 years of age [1], 2017. As defined by the World Bank classification; Singapore is a high‐income country (HIC). The prevalence of CP in HIC is 1.5–1.6 per 1000 live births [2]. People with CP may be affected by weakness, abnormal muscle tone such as spasticity, involuntary movement, poor motor control and contractures [3]. These impairments can affect functional ability and participation [3].

The management of CP should be integrated and multidisciplinary [4]. It may include rehabilitative, pharmacological and surgical interventions [5]. Electrical stimulation, such as transcutaneous electrical nerve stimulation (TENS) and neuromuscular electrical stimulation (NMES) have shown promising results in reducing muscle tone, increasing muscle strength, and improving motor function [6, 7]. TENS is the application of low‐intensity electrical stimulation primarily targeting nerves. It is more commonly used for pain management, however there is some evidence to show that it can potentially reduce spasticity by interfering with nerve signals that contribute to muscle contraction [8]. NMES uses electrical stimulation to activate specific muscles to improve strength. This concurrently relaxes antagonist muscle groups, thereby reducing spasticity and enhancing functional movement [6, 9].

**Figure 1:**
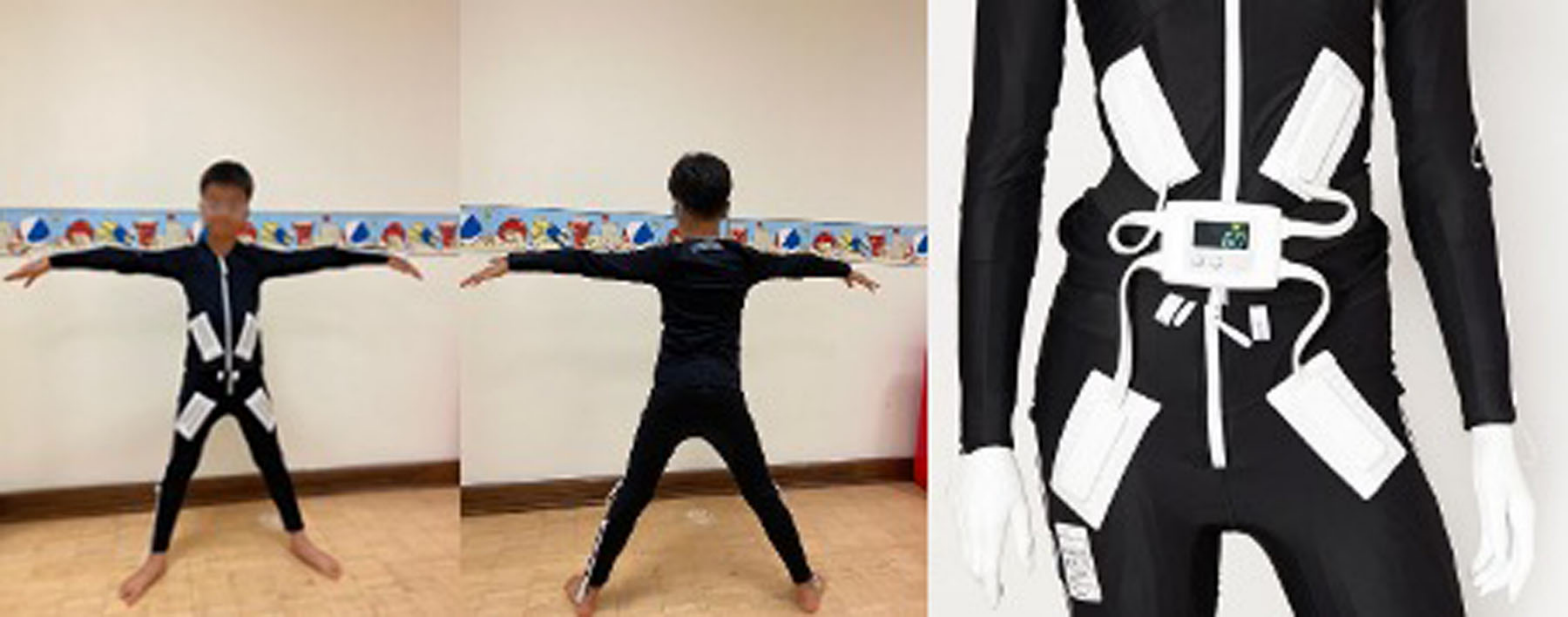
Mollii suit garment.

Considering the growing evidence for TENS and NMES in the care of individuals with CP, the Expopulse Mollii Suit (known as Mollii Suit) is a novel approach that incorporates electrical stimulation in a whole‐body garment [10]. It has 58 imbedded electrodes, which are individually programmed to stimulate selected muscles using a control unit. The control unit is attached to the suit magnetically and is worn at the waist (Figure 1). The Mollii Suit has a different mechanism of action from TENS and NMES. The suit purportedly reduces spasticity through the concept of reciprocal inhibition via subthreshold electrical stimulation to the antagonist of spastic muscles [10]. The electrodes activate inhibitory 1a interneurons in the spinal cord which act to reduce the excitability of the spastic muscle's motor neuron [10].

There are only five published studies to our knowledge that have investigated the use of the Mollii Suit in children with CP [11, 12, 13, 14, 15]. The quality of evidence was weak. The studies had limitations such as small or heterogeneous sample size (6–15 participants), lack of control group and poor reproducibility. Three studies investigated functional ability and another two studied the qualitative experience of using the suit. Bakaniene, Urbonaviciene, Janaviciute & Prasauskiene [13] found an improvement in gross motor skills (measured by the Gross Motor Function Measure‐88 [GMFM‐88]) although the study was not powered adequately to show significant difference. There was no superior efficacy on gross motor ability using the Mollii Suit over conventional care. Flodstrom et al [15] found that three out of six children had a significant difference in the Canadian Occupational Performance Measure. Parents reported that they noticed some improvements however, there were some challenges reported too. Parents explained that as assistance was required to don the suit, burden of care for the parent was increased [12, 14].

The Mollii Suit could be appealing to families that desire an alternative adjunct home‐based therapy for their children with CP. Considering the above potential benefits, we aim to study the impact of Mollii Suit therapy in improving gait and function in children with CP. The hypothesis is that the use of the Mollii Suit as an adjunctive therapy in children with CP will result in significant improvements in gait and motor function compared to standard care alone, providing a viable and appealing therapeutic option for families. We would like to evaluate if there is any immediate response after using the suit and if there is an carry over effect one month later.

## 2 Methodology

### 2.1 Study Design

This is a single‐center prospective study conducted at KK Women's and Children's Hospital (KKH), Singapore. The study was approved by SingHealth Centralised Institution Review Board (Reference number: 2020/2150) and registered on ClinicalTrials.gov (NCT04715334). Written informed consent was obtained from each participant's legal guardian. The Template for Intervention Description and Replication has been used for replicability (Supporting Information SI) [16].

### 2.2 Subjects

We included individuals aged 4–18 years with spastic CP and Gross Motor Function Classification System level I–III who attended our outpatient clinics January 2021 to March 2022. Exclusion criteria were as follows: (1) implanted electrical stimulatory device; (2) implanted ferromagnetic devices such as programmable shunt; (3) active history of cardiovascular disease, infectious disease, malignancy, fever, pregnancy, rash or skin disease; (4) Botulinum therapy or soft tissue release surgery within the last 6 months; (5) change in oral medication for spasticity within the last 1 month; and (6) children needed to be able to complete the outcome measures (i.e., gait study and GMFM‐88). Both parental consent and assent from the children were taken.

### 2.3 Materials

The Mollii Suit (Figure 1) was designed by Exoneural Network AB, (https://www.ottobock.com/en-ex/exopulse-b2c). The Mollii Suit has been demonstrated to be safe for use [11, 15]; Nordstrom & Pellwitz, 2021 [14].

### 2.4 Intervention

The Mollii Suit was fitted and programmed for each participant by vendor‐accredited physiotherapists at KKH, with the programming advised by the vendor's appointed engineer. Electrodes are activated via a control unit which is attached to the suit. The program is set from computer software and transferred to the control unit. The Mollii Suit program parameters were customized to the participant's age and distribution of spasticity and weakness (please refer to Table 1).

**Table 1:** Generic Mollii Suit Program developed by Frederik Lundavist (Guide provided by Exoneural Network AB, now part of the Ottobock portfolio).

1	**Age**	
	Contrary to standardized intensity settings in adults, the patient's age is used as a guide in determining the stimulation parameters in a pediatric patient. Maximum intensity equivalent to age is chosen for (a) foot dorsiflexion and plantar flexion; (b) knee extension c) hip abduction and hip extension. For example, age = 12, intensity = 12. 2/3 of maximum intensity for age is chosen for (a) knee flexion; (b) hip flexion; (c) hip adduction; (d) hand extension; (e) shoulder external rotation 1/3 of maximum intensity for age is chosen for (a) trunk extension; (b) elbow extension; (c) hand flexion; (d) elbow flexion; (e) shoulder internal rotation/adduction 1/4 of maximum intensity for age is chosen for (a) trunk flexion; (b) upper thoracic extension; (c) shoulder abductors; (d) neck flexion	Standardized intensity level settings for any adult: 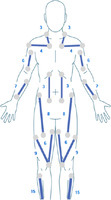 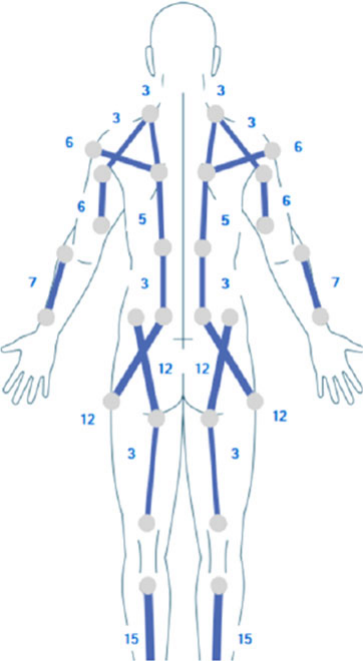
2	**Diagnosis**	
	The type of CP determines which electrodes are activated and at what intensity (e.g., spastic, ataxic, dystonic or athetoid). The image demonstrates the location of muscles to be activated for spastic diplegia. For hemiplegia, the muscles activated would be the same but only one side of the body.	Muscles to activate for spastic diplegia (bilateral lower limbs affected) (concept of reciprocal inhibition): 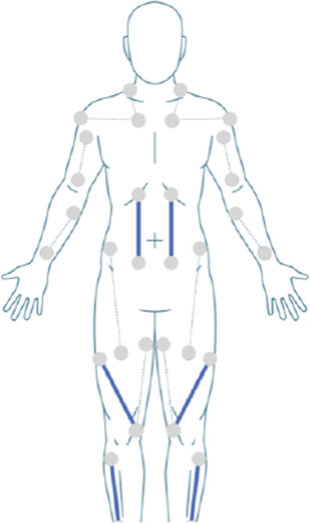 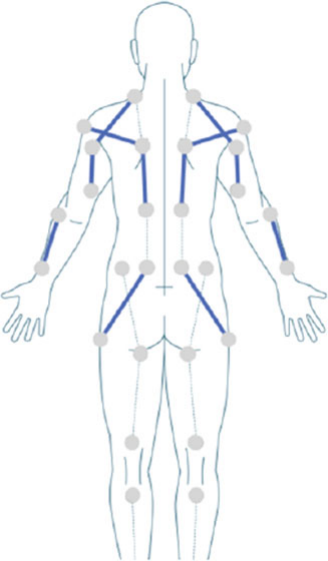
3	**Extremities/side of the body affected**	
	The number and side of affected quadrants determines which electrodes are activated (e.g., bilateral or unilateral CP).	

Participants wore the suit in a home setting for 60 min a day for 4 weeks. There were no instructions or requirements during the 60 min. The children could choose to do any activity they liked, including sitting and lying down. Throughout the study period, they were allowed to continue with their usual daily routine, which might include physiotherapy and exercise. This duration was chosen based on prior research and experience by developers during their development phase [17].

### 2.5 Outcome Measures

Outcome measures were taken face to face at baseline (preintervention), immediately post intervention and 1‐month postintervention 1 (Table 2). Two different time points were assessed to review if there was any effect immediately after wearing the suit and if there was any residual response 1‐month thereafter.

**Table 2:** Summary of study visits.

VISIT 1	Preintervention assessments (baseline assessment) Part 1: – Instrumented 3‐dimensional gait study – Tardieu scoring – Questionnaires (include EQ‐5D)
VISIT 2 (within 1 week of Visit 1):	Preintervention assessments (baseline assessment) Part 2: – GMFM – Collection of Mollii suit, settings for Mollii suit Signing of loan agreement
HOME 4 WEEKS	Patient will wear Molli suit at home 1 h daily for 4 weeks.
	Patient/parent keep log logs of compliance use, pain and adverse effects.
VISIT 3*	Return Mollii Suit
	Post‐intervention assessments: – Instrumented 3‐dimensional(3D) gait study – Tardieu scoring – Questionnaires (include EQ‐5D) – GMFM
VISIT 4 (1 MONTH postintervention)	1 month postintervention assessments: – Questionnaires (include EQ‐5D) – GMFM

*Note:* No 3D gait study was performed at Visit 4, this is due to the lack of funding available from the grant to sponsor a third 3D gait study.

### 2.6 Gait Outcome Measures

The 3D gait analysis required participants to walk on a 10‐meter walkway with two force‐plates. The researcher placed skin markers placed on the participant's body, following the Plug‐in marker placement model [18]. The patient walked barefoot repeatedly along the walkway until 6–8 successful clean strikes were made on the force plate per foot. An average of 3 was taken for data analysis. If the child used a walking aid at home, their gait assessment was done with their usual walking aid. The VICON infrared camera system was used to capture the trials, after which, raw data was processed using NEXUS software (v2.0).

### 2.7 Gait Parameters

Gait Profile Score (GPS), Gait Deviation Index (GDI), cadence (step/min) and walking speed (m/s) were assessed. Gait parameters are assessed separately for right and left lower limbs. Due to the large about of information generated by gait analysis, a number of indices and scores have been designed to condense complex data into easier interpretation [19].

GPS is a single index measure that summarizes the overall deviation of kinematic gait data relative to normative data. It is composed of the Gait Variable Scores, which are calculated from nine key kinematic gait variables. It represents the root mean square difference between a particular trial and average data with no gait pathology. It ranges from 0 to 30 –the higher the score, the more abnormal gait pattern [19].

GDI provides a numerical value that expresses overall gait pathology (score ranges from 0 to 100). The score of 100 is a typical gait pattern [20].

### 2.8 Functional Outcome Measures

The GMFM‐88 is an assessment tool designed and validated to measure changes in gross motor function in children with CP. There are five subdomains: (a) lying and rolling, (b) sitting, (c) crawling and kneeling), (d) standing and (e) walking, running and jumping. The higher the GMFM score, the better the function [21].

Other outcomes of function included modified Tardieu scoring (measures spasticity), EQ‐5D and EQ‐5D‐Y. The EQ‐5D was completed by the parent based on what they think the child felt and the EQ‐5D‐Y was completed by children who were able to. The EQ‐5D‐Y has been widely used to describe and value health in younger populations [22]. It is a descriptive system which measures health across five dimensions and a general rating of health on a visual analog scale (VAS) of 0 (worst health) to 100 (best health); domains include *Mobility* (walking about), *Looking After Myself* (washing and dressing), *Doing Usual Activities* (going to school, hobbies, sports, playing, doing things with family or friends), *Having Pain or Discomfort* and *Feeling Worried, Sad or Unhappy* (ER Foundation, 2019).

### 2.9 Others

Compliance rate and pain score (Wong‐Baker Faces Scale or Visual Analog Scale depending on participant's age) was obtained from the daily logs of suit usage completed by the participants. Potential adverse events (discomfort, tingling, tightness of suit, temporary marks from the electrodes or skin redness) were written on the consent form and participants were provided with a copy. Study participants and their parents were advised to immediately report any major adverse event to the study team. Qualitative information on patient satisfaction and feedback were also collected using a specially designed questionnaire (can be found in Supporting Information S1: Annex I–VII)

A summary of the study visits and outcome measures can be found in Table 2 below.

### 2.10 Statistical Analysis

Statistical analyses were performed using SPSS v. 24.0 (SPSS Inc. Chicago.). Tests of normal distribution were done and data was found to be normally distributed. Paired t‐test was performed on the change in outcomes measured between two time points. The GMFM and the questionnaires had three time points, paired *t*‐test was used to measure between pre and post and pre and 1‐month post. Sample size of 20 was determined based on a two‐sided paired *t*‐test at 80% power and significance level of 0.05 to detect a minimal clinically important difference of 1.6 in GPS. This was originally defined in a sample of children with CP [23]. *p* < 0.05 was considered statistically significant. Although there is a high amount of variability with a small sample size, the nature of CP is that there is a huge amount of variability. The results represent a typical cohort of children with CP.

## 3 Results

Twenty children of median age 7 years old (range: 4–16) were recruited. Parents completed the journal for compliance rate and pain score together with their children. Compliance rate was high at 95%, 19 out of 20 completed all 28 days of wearing the suit. One child with concomitant autistic spectrum disorder dropped out due to intolerability of suit. Another child was unable to complete post‐intervention gait study due to traveling overseas for a procedure.

Table 3 demonstrates the demographics of the participants.

**Table 3:** Basic demographics (*n* = 20).

Demographic variables	*n* (%)
**Sex**	
Male	11 (55)
Female	9 (45)
**Ethnic group**	
Chinese	13 (65)
Malay	3 (15)
Indian	2 (10)
Other	2 (10)
**Age**	
2–6 years (preschool)	6 (30)
7–16 years (school)	14 (70)
**Topography**	
Bilateral	17 (85)
Unilateral	3 (15)
(2 right‐sided, 1 left‐sided)	
**GMFCS**	
I	3 (15)
II	11 (55)
III	6 (30)

## 4 Gait Outcome Measures

### 4.1 Gait Outcome Measures Were Analysed As

Postintervention, there was a significant change in GPS left, right and overall. However, GPS log difference on left, right and overall did not reach minimally important clinical difference (MCID) of 1.6.

There was no significant change in mean GDI left and right pre and postintervention. There was an improving trend in temporo‐spatial parameters although it did not reach statistical significance. Table 4 summarizes the change in mean and standard deviation values for gait parameters pre and postintervention.

**Table 4:** Gait parameters at baseline and postintervention (*n* = 18).

Gait variable	Baseline ± SD	Postintervention ± SD	95% CI (Interval of the lower to upper difference)	*p* value
GPS Overall	16.68 ± 4.31	20.80 ± 9.40	−7.012 to −1.234	< 0.01*
GPS left	14.40 ± 4.34	17.69 ± 9.12	−6.16 to −0.42	0.027*
GPS right	16.81 ± 4.74	20.90 ± 8.72	−6.82 to −1.36	0.006*
GDI left	66.59 ± 12.47	62.36 ± 17.0	−0.3 to 8.77	0.07
GDI right	65.93 ± 12.79	64.80 ± 14.21	−3.27 to 5.52	0.60
Cadence left (step/min)	103.44 ± 38.25	107.35 ± 32.19	−16.22 to 8.39	0.51
Cadence right (step/min)	102.93 ± 35.55	108.75 ± 33.81	17.60 to 5.965	0.31
Walking speed left (m/s)	0.70 ± 0.36	0.74 ± 0.36	−0.11 to 0.04	0.29
Walking speed right (m/s)	0.69 ± 0.36	0.73 ± 0.36	−0.12 to 0.03	0.21

### 4.2 Functional Outcome Measures

#### 4.2.1 GMFM‐88

**Figure 2:**
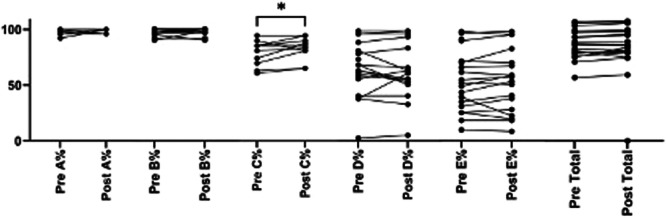
GMFM‐88 scores pre and postintervention (*n* = 19).

GMFM A (*Lying and Rolling*), B (*Sitting*), C (*Crawling and Kneeling*), E (*Walking*) and Total improved after 1‐month use of suit. However, only mean GMFM Domain C improved significantly from 88.47% ± 11.42% to 91.73% ± 9.54% (95% CI: 0.44–6.07, *p* = 0.026) (refer Figure 2). Results were no longer significant at 1‐month thereafter.

#### 4.2.2 EQ‐5D‐Y and EQ‐5D (Parent Reported)

Thirteenchildren were able to provide EQ‐5D‐Y data. After 1 month of suit usage, all aspects of EQ‐5D‐Y improved; *usual activity* improved significantly from mean of 1.46 ± 0.52 to 1.15 ± 0.38 (95% CI: 0.02–0.60, *p* = 0.040). Results were no longer significant at 1‐month thereafter. A total of 19 parents completed the EQ‐5D. Parent‐reported EQ‐5D did not improve.

Tables 1, 2, 3 in Supporting Information S1: Annex VIII show the GMFM‐88 and EQ‐5D (y) and EQ‐5D (parent) results.

### 4.3 Tardieu Scores

Spasticity was measured in the gastrocnemius, hamstring and quadriceps muscles (refer Table 4). A smaller difference between R2 and R1 demonstrates less spasticity (25). Only the hamstring muscles for the right lower limb had a significant improvement in spasticity. The rest of the results did not attain statistical significance. Results can be seen in Table 5.

**Table 5:** Tardieu scores pre and postintervention.

Muscle	Right lower limb			Left lower limb		
	Pre (difference between R2 and R1)	Post (difference between R2 and R1)	*p* value	Pre (difference between R2 and R1)	Post (difference between R2 and R1)	*p* value
Gastrocnemius (Right *n* = 15, left *n* = 14)	14.73 ± 7.81	13.53 ± 6.90	0.54	10.86 ± 6.26	14.93 ± 8.32	0.08
Hamstring (*n* = *18)*	16.17 ± 8.82	10.55 ± 9.25	0.03*	13.28 ± 10.02	11.39 ± 11.14	0.43
Quadriceps (*n* = 18)	31.94 ± 36.18	45.94 ± 50.95	0.31	42.28 ± 44.09	34.72 ± 42.65	0.53

*Note:* Value presented as Mean ± SD.

### 4.4 Adverse Events

Nil major adverse event was reported. Twelve (60%) participants experienced transient pain/discomfort while using the suit, with the mean pain score of 0.75 ± 1.47 (out of maximum 10). Only one participant complained of transient stiffness in the arm and one patient had a transient red mark.

### 4.5 Overall Experience and Patient/Caregiver Satisfaction

Parents filled out a questionnaire regarding satisfaction of the Mollii Suit (excellent, good, fair and poor). The majority of parents and participants (*n* = 15, 75%) perceived excellent or good experience and reported that the suit helped in general functional ability. However, despite the good experience parents and children gave feedback that the use of the suit was that it was difficult to apply (*n* = 15, 75%), tight or warm to wear in the local climate (*n* = 11, 73.3%).

## 5 Discussion

This study evaluated the impact of the Mollii Suit on gait and function in children with CP. The results demonstrated that although GPS had a statistically significant change, it did not reach MCID as reflected in Table 4. However, gross motor function in terms of *crawling and kneeling* and *usual activity* as reported by child in EQ‐5D‐Y improved significantly after intervention but were no longer significant after 1 month. There were nil major adverse events related to the use of the suit. Two different time points were assessed to review if there was any effect immediately after wearing the suit and if there was any residual response 1‐month therafter.

The study results showed that the intervention did not bring about clinical improvement in gait profile. Based on the proposed theory that the Mollii Suit is able to reduce spasticity via reciprocal inhibition, we postulated that reduction of spasticity revealed underlying lower limb weakness, which may contribute to gait instability. This phenomenon has been reported previously after Botulinum toxin injections and surgical procedures [5, 24]. To see an improvement in gait quality, a much longer intervention duration may be required to allow for strengthening of the muscles.

In our study, the increase in GPS did not reach MCID. It should also be noted that the GPS and GDI are measures of gait quality during straight line walking on a clear level surface within a gait analysis laboratory [23]. Other clinically important outcome measures, particularly in more real‐world environments, should also be considered, for example the Functional Ambulation Profile (a composite score derived from walking tests, obstacle negotiation and stair climbing) In our study, there was an improving trend in temporo‐spatial parameters, such as increased cadence and gait speed. These parameters are helpful in determining community ambulation (e.g., crossing the road). However, this study is not powered to detect a significant change in these temporo‐spatial variables. We gathered temporo‐spatial parameters during straight line walking in the context of a gait lab. These parameters could be translated better than GPS or GDI if tested in the community, however, as the temporo‐spatial parameters were not tested in the community, so may carry some of the same limitations as GPS and GDI.

Improvement in GMFM Domain C (*crawling and kneeling*) and EQ‐5D‐Y (*usual activity)* were statistically significant. It is hypothesized that the reduction of spasticity may allow for an improvement in gross motor skills and selective muscle control [13]. However, research has shown that spasticity only partially explains reduced functional skills and poor gait pattern in children with CP. It has been suggested that weakness may in fact account for more disability than spasticity, indicating that time to improve muscle strength is needed [25].

In our study, participants continued their usual standard of care during the use of the Mollii Suit. We do not have any information on how many of the participants participated in physiotherapy or strengthening during the course of the 1 month of intervention. Findings from reviews suggested the use of electrical stimulation‐assisted therapy concurrent with strengthening exercises improved strength and mobility in individuals with spastic CP [26]. Thus, an active strengthening program to work on underlying weak muscles after changes in spasticity may be needed to bring about improvement in gait quality and other function. Improvement in outcome measures were no longer significant 1 month after intervention. Longer intervention studies are needed to ascertain potential carry over effect.

Compliance and satisfaction was generally high with 75% of participants stating that they perceived the suit to be excellent or good. The Mollii Suit may be a useful adjunct home‐based therapy for children with spastic CP, however it may be of limited use in a climate such as Singapore, which is hot and humid. Parents have reported that the suit was difficult to apply and was warm. It may be more suited for a cooler climate.

## 6 Strengths and Limitations

This is the first study that uses instrumented 3D gait analysis to objectively measure change in kinematic and temporo‐spatial parameters in gait. This study also evaluated clinically important patient‐centric functional measures. Compliance rate for intervention was also high. This study is also reproducible and sample sizes were larger than previous studies. Limitations are that it was an unblinded study with no control group. This study was a feasibility study to determine if any response and if able to tolerate the Molii Suit in Singapore. We did a prepostintervention design as natural disease progression in CP does not tend to change rapidly over 1 month; therefore, any changes were likely related to the intervention. The study design may raise the possibilities of placebo effect, performance bias, and recall bias, all of which could influence the results. The small sample size potentially reduces generalizability to the larger CP population. During the study, participants continued their usual standard of care, so the physical therapy program was not standardized.

CP is heterogenous with different clinical types, comorbidities, brain imaging patterns, causes and genetic variants. This sample is representative of a typical cohort of children with CP. The ethnic group distribution and topography of CP is similar to our local CP cohort. We took into consideration our heterogenous sample and wide age range by doing pre‐post testing and paired T tests. A weakness of the study is that there was no third 3D gait study performed at the 4th Visit, there were not enough funds from the grant to support a third 3D gait study.

The authors affirm that this manuscript is an honest, accurate, and transparent account of the study being reported; that no important aspects of the study have been omitted; and that any discrepancies from the study as planned (and, if relevant, registered) have been explained.

## 7 Conclusion

The Mollii Suit made positive changes in gait and function in children with spastic CP immediately after the 4 week intervention period. However, further studies are required to determine its broader impact.

## Author Contributions

**Lindsey Jean Ross Weller:** investigation, writing – original draft; methodology, writing – review and editing, formal analysis, data curation. **Shelly‐Anne Marie Sherwood:** investigation, writing – original draft, methodology, writing – review and editing, data curation, formal analysis. **Shin Huey Ng:** investigation, writing – original draft, writing – review and editing, formal analysis, data curation. **Maheswari Vellaichamy:** investigation, writing – original draft, methodology, writing – review and editing, data curation, formal analysis. **Asila Alia Noordin:** investigation, writing – original draft, methodology, writing – review and editing, data curation, formal analysis. **Ling Ying Tan:** investigation, writing – original draft, methodology, writing – review and editing, formal analysis, data curation. **Arjandas Mahadev:** investigation, writing – original draft, writing – review and editing, formal analysis, data curation. **Tong Hong Yeo:** investigation, writing – original draft, writing – review and editing, data curation, formal analysis. **Zhi Min Ng:** conceptualization, investigation, funding acquisition, writing – original draft, methodology, writing – review and editing, formal analysis, data curation.

## Ethics Statement

The study was approved by SingHealth Centralised Institution Review Board (Reference number: 2020/2150) and registered on ClinicalTrials.gov (NCT04715334).

## Consent

Written informed consent was obtained from each participant's legal guardian and assent was taken from each participant.

## Conflicts of Interest

The authors have stated that they had the Molli Suit garments donated by Exoneural Network AB, which is now under the portfolio of Ottobock.

## Transparency Statement

The lead author Lindsey Jean Ross Weller affirms that this manuscript is an honest, accurate, and transparent account of the study being reported; that no important aspects of the study have been omitted; and that any discrepancies from the study as planned (and, if relevant, registered) have been explained.

## Supporting information

Supporting information.

## Data Availability

The data that support the findings of this study are available from the corresponding author upon reasonable request.
