# Supplementary material for: Can External Neuromodulation Garments Improve Gait and Function in Children With Cerebral Palsy? A Prospective Single‐Arm Study
Source: Health Sci Rep. 2025 Mar 23;8(3):e70566. doi: 10.1002/hsr2.70566 (PMC11930893; doi:10.1002/hsr2.70566)
Supplement: Supplementary file 1 — Supporting information. [file HSR2-8-e70566-s001.docx]

# Annex I: Demographic Questionnaires - Child/teenager

1. The following questions ask about your health.

| Under each heading, please tick **ONE** box that best describes your health **TODAY**. | |
| --- | --- |
| **Mobility (walking about)** | |
| I have no problem moving about. | 🞏 |
| I have some problems walking about. | 🞏 |
| I have a lot of problem walking about. | 🞏 |
| **Looking after myself** | |
| I have no problem washing or dressing myself. | 🞏 |
| I have some problem washing or dressing myself. | 🞏 |
| I have a lot of problems washing or dressing myself. | 🞏 |
| **Doing usual activities (for example, go to school, hobbies, sports, play, do things with family and friends)** | |
| I have no problem doing my usual activities. | 🞏 |
| I have some problem doing my usual activities. | 🞏 |
| I have a lot of problems doing my usual activities. | 🞏 |
| **Having pain or discomfort** | |
| I have no pain or discomfort. | 🞏 |
| I have some pain or discomfort. | 🞏 |
| I have a lot of pain or discomfort. | 🞏 |
| **Feeling worried, sad or unhappy** | |
| I am not worried, sad or unhappy. | 🞏 |
| I am a bit worried, sad or unhappy. | 🞏 |
| I am very worried, sad or unhappy. | 🞏 |
| We would like to know how good or bad your health is TODAY.  This line is numbered from 0 to 100.  100 means the best health you can imagine.  0 means the worst health you can imagine.  Please mark an X on the line that shows how good or bad your health is TODAY. |  |

2. Please let us know what you think about wearing the Mollii suit at home for 4 weeks.

____________________________________________________________________________________________________________________________________________________________________________________________________________________________________________________________________________________________________________________

## Annex II: Demographic Questionnaire – PARENT

| 1. Housing/ care arrangement | | | | | | | |
| --- | --- | --- | --- | --- | --- | --- | --- |
| Housing ownership | | 🞏Fully owned 🞏Loaned/Mortgaged 🞏Living with parents 🞏Rented 🞏Company residence | | | | | |
| Housing type | | 🞏HDB 🞏Condominium 🞏Landed | | | | | |
| Number of bedrooms | | 🞏1      🞏2      🞏3      🞏4 🞏more than 4 | | | | | |
| Combined family income (per month) | | _____________ | | | | | |
| No. of household members in the house: | | ______ child/children under 18 years old  ______ adults (18 years and above)  ______ domestic worker/s | | | | | |
| Financial assistance scheme | | 🞏Yes    🞏No  If yes, specify: ____________________________ | | | | | |
| Main caregiver (≥1 tick is possible): | | 🞏Mother 🞏Father 🞏Grandparent 🞏Helper 🞏Other: ______________ | | | | | |
| 2. ICF | 0: No problem/ difficulty  1: Mild problem/ difficulty (present <25% time)  2: Moderate problem/difficulty (present 25-50% time)  3: Severe problem/ difficulty (present >50% time)  4: Complete problem/ difficulty (present >95% time) | | | | | | |
| **BODY FUNCTIONS** | | | **0** | **1** | **2** | **3** | **4** |
| Does your child sleep enough? | | | 🞏 | 🞏 | 🞏 | 🞏 | 🞏 |
| Does your child have problem falling asleep? | | | 🞏 | 🞏 | 🞏 | 🞏 | 🞏 |
| Does your child have problem staying asleep? | | | 🞏 | 🞏 | 🞏 | 🞏 | 🞏 |
| Does your child have good quality of sleep/ feel rested? | | | 🞏 | 🞏 | 🞏 | 🞏 | 🞏 |
| Does your child have generalised pain? | | | 🞏 | 🞏 | 🞏 | 🞏 | 🞏 |
| Does your child have problem with his/her tone of all muscles of body | | | 🞏 | 🞏 | 🞏 | 🞏 | 🞏 |
| Does your child have difficulty with involuntary contractions of muscles? | | | 🞏 | 🞏 | 🞏 | 🞏 | 🞏 |
| **ACTIVITIES & PARTICIPATION** | | | **0** | **1** | **2** | **3** | **4** |
| Does your child have difficulty maintaining a sitting position (supported/ independent)? | | | 🞏 | 🞏 | 🞏 | 🞏 | 🞏 |
| Is it easy to transfer your child? | | | 🞏 | 🞏 | 🞏 | 🞏 | 🞏 |
| Is it easy to perform toileting needs of your child? | | | 🞏 | 🞏 | 🞏 | 🞏 | 🞏 |
| Is there difficulty/ problem for your child to make basic interpersonal interactions? | | | 🞏 | 🞏 | 🞏 | 🞏 | 🞏 |
| Is there difficulty/ problem for your child to make family relationships | | | 🞏 | 🞏 | 🞏 | 🞏 | 🞏 |

3. Which of the following mobility devices does your child use to get around?

€ No device € Quadsticks

€ Crutches € Walker

€ Manual wheelchair € Motorised wheelchair

€ Other (please describe) _____________________________________

4. Your child’s health TODAY

| Under each heading, please tick **ONE** box that best describes your child’s health **TODAY**. | |
| --- | --- |
| **Mobility (moving about)** | |
| My child has no problem moving about. | 🞏 |
| My child has some problems moving about. | 🞏 |
| My child has a lot of problem moving about. | 🞏 |
| **Looking after self** | |
| My child has no problem washing or dressing him/herself. | 🞏 |
| My child has some problem washing or dressing him/herself. | 🞏 |
| My child has a lot of problems washing or dressing him/herself. | 🞏 |
| **Doing usual activities (for example, go to school, hobbies, sports, play, do things with family and friends)** | |
| My child has no problem doing my usual activities. | 🞏 |
| My child has some problem doing my usual activities. | 🞏 |
| My child has a lot of problems doing my usual activities. | 🞏 |
| **Having pain or discomfort** | |
| My child has no pain or discomfort. | 🞏 |
| My child has some pain or discomfort. | 🞏 |
| My child has a lot of pain or discomfort. | 🞏 |
| **Feeling worried, sad or unhappy** | |
| My child is not worried, sad or unhappy. | 🞏 |
| My child is a bit worried, sad or unhappy. | 🞏 |
| My child is very worried, sad or unhappy. | 🞏 |
| We would like to know how good or bad your child’s health is TODAY.  This line is numbered from 0 to 100.  100 means the best health you can imagine.  0 means the worst health you can imagine.  Please mark an **X** on the line that shows how good or bad your child’s health is **TODAY**. |  |

The next question relates to your child’s previous medication/management.

5. Does your child have (or has he/she ever had) medical intervention to manage spasticity? Such as:

Botulinum toxin (Botox)______________________________________________­­­­­_______

Baclofen___________________________________________________________________

Other, please name__________________________________________________________

6. Has your child used any form of Transcutaneous Electrical Stimulation before? Yes/no

Please describe ________________________________________________________________________________________________________________________________________________________________

7. Please tell us why you and your child are interested in participating in this research project about the Mollii suit.

________________________________________________________________________________________________________________________________________________________________________________________________________________________________________________

8. Please tell us what you hope the Mollii Suit will assist your child with.

________________________________________________________________________________________________________________________________________________________________________________________________________________________________________________________________________________________________________________________________

9. Please tell us how much you think you will pay for the rental of the Mollii Suit over 4 weeks.

________________________________________________________________________________________________________________________________________________________________________________________________________________________________________________

­

## Annex III: Study Compliance Diary

This purpose of this Study Diary to gather valuable information to assist us with our evaluation and assessment of the Mollii suit study. Please keep this diary in a handy place near or with the Mollii suit and record the date and time when you help your child wearing the Mollii suit at home.

**Every time** your child wears the Mollii suit, please record the date and how long your child wears the suit in the following table.

| **Date** | **How long your child wear the Mollii suit? (minutes)** | **Any reaction or complaint from your child while wearing the suit?** | **PAIN SCORE**  **(refer figure below)** |
| --- | --- | --- | --- |
|  |  |  |  |
|  |  |  |  |
|  |  |  |  |
|  |  |  |  |
|  |  |  |  |
|  |  |  |  |

**
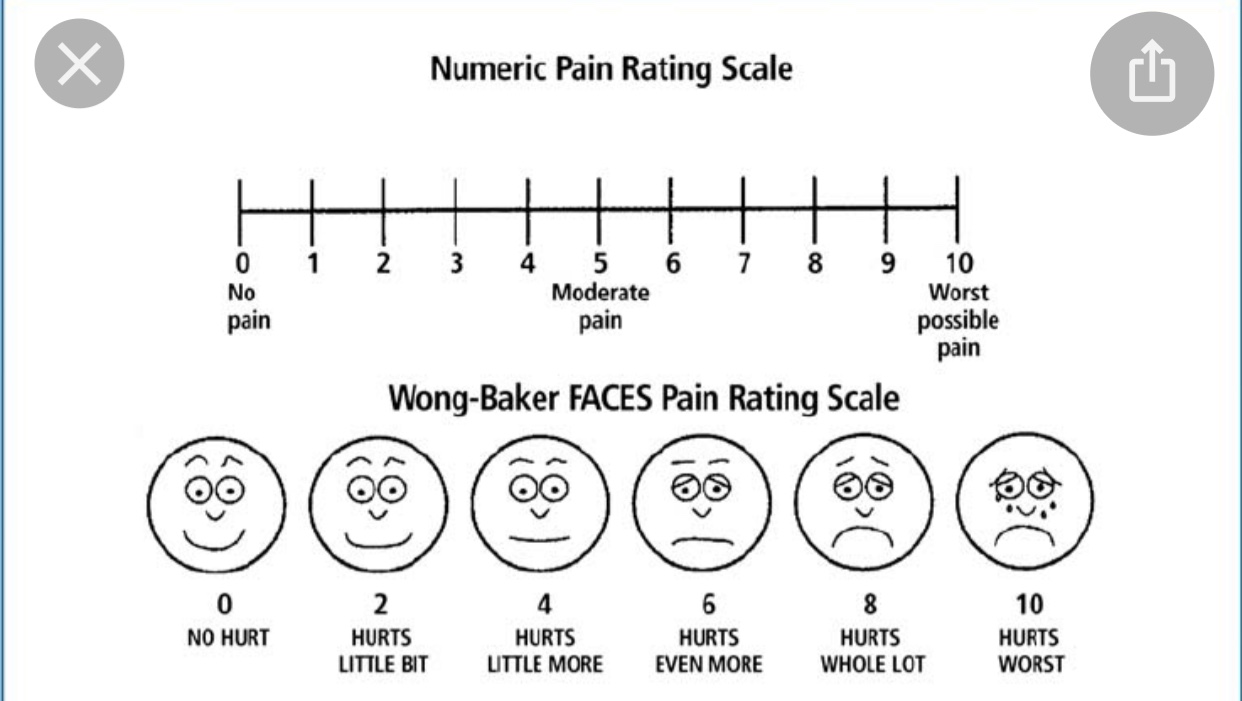
**

| **Date** | | **How long your child wear the Mollii suit? (minutes)** | | **Any reaction or complaint from your child while wearing the suit?** | **PAIN SCORE**  **(refer figure below)** | |
| --- | --- | --- | --- | --- | --- | --- |
|  | |  | |  |  | |
|  | |  | |  |  | |
|  | |  | |  |  | |
|  | |  | |  |  | |
|  | |  | |  |  | |
|  | |  | |  |  | |
|  | |  | |  |  | |
|  | |  | |  |  | |
|  | |  | |  |  | |
| **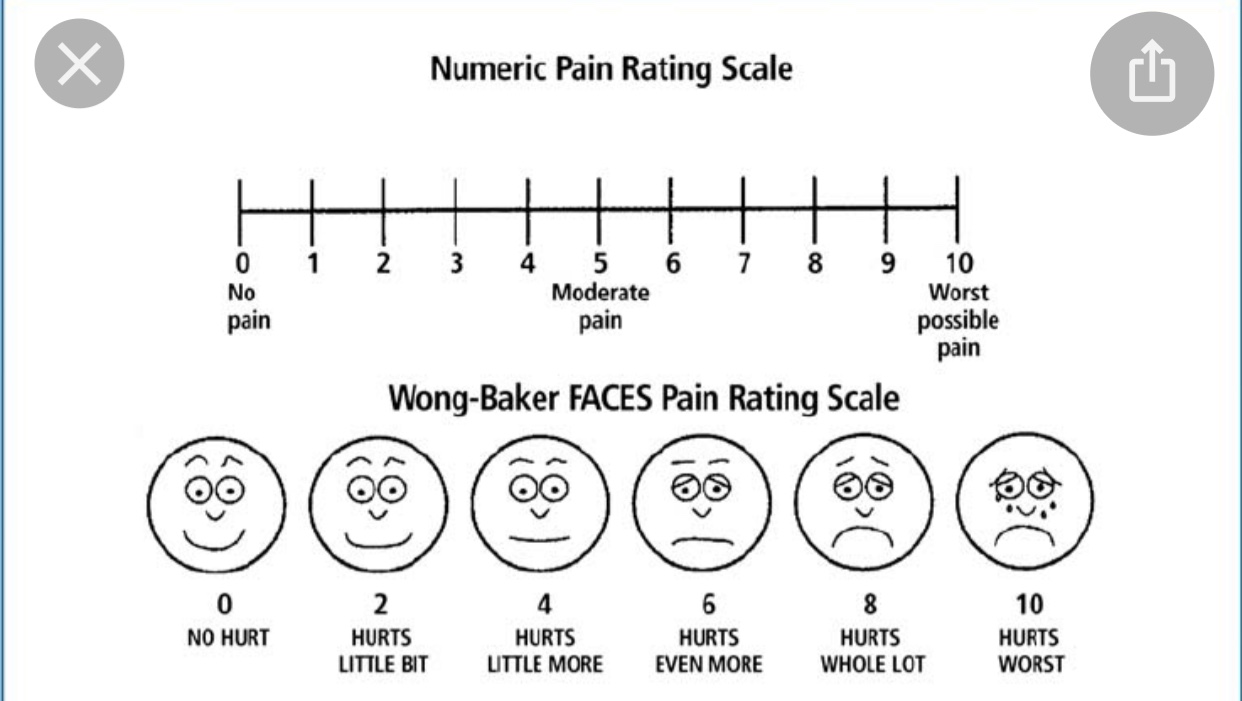** | | | | | | |
| **Date** | | **How long your child wear the Mollii suit? (minutes)** | | **Any reaction or complaint from your child while wearing the suit?** | **PAIN SCORE**  **(refer figure below)** | |
|  | |  | |  |  | |
|  | |  | |  |  | |
|  | |  | |  |  | |
|  | |  | |  |  | |
|  | |  | |  |  | |
|  | |  | |  |  | |
|  | |  | |  |  | |
|  | |  | |  |  | |
|  | |  | |  |  | |
| **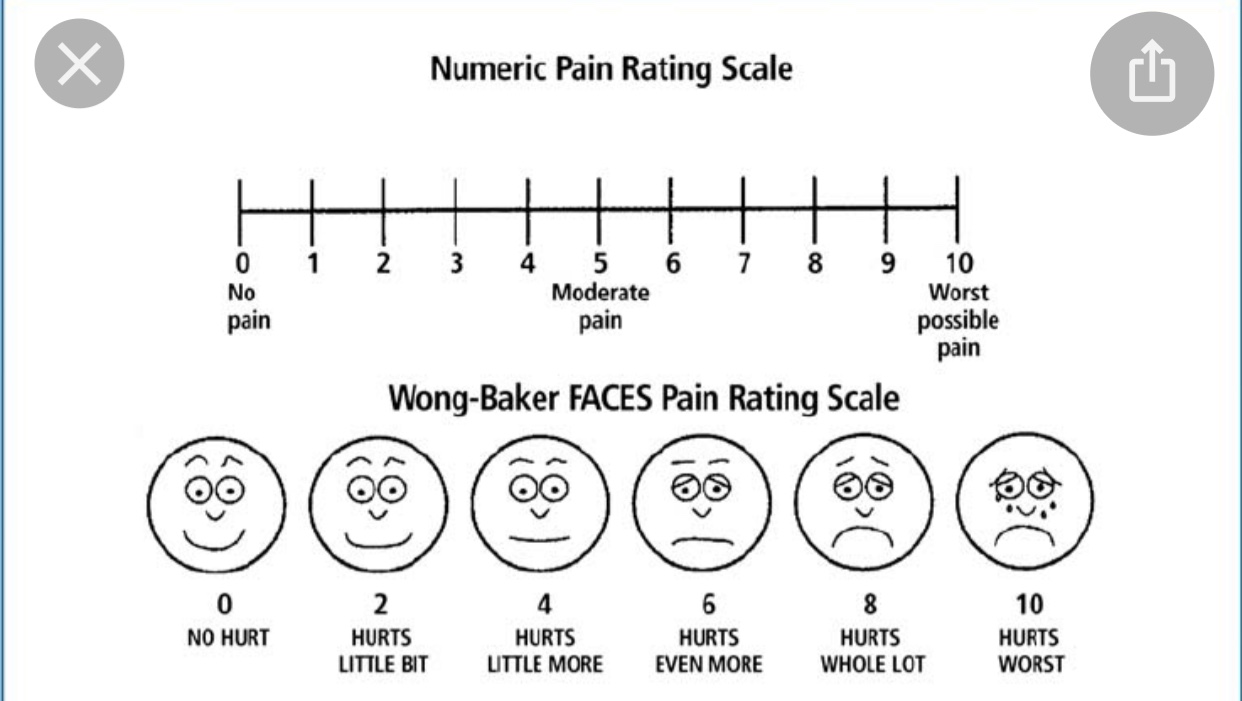** | | | | | | |
|  |  | |  | | |  |
| **Date** | **How long your child wear the Mollii suit? (minutes)** | | **Any reaction or complaint from your child while wearing the suit?** | | | **PAIN SCORE**  **(refer figure below)** |
|  |  | |  | | |  |
|  |  | |  | | |  |
|  |  | |  | | |  |
|  |  | |  | | |  |
|  |  | |  | | |  |
|  |  | |  | | |  |
|  |  | |  | | |  |
|  |  | |  | | |  |
|  |  | |  | | |  |
| **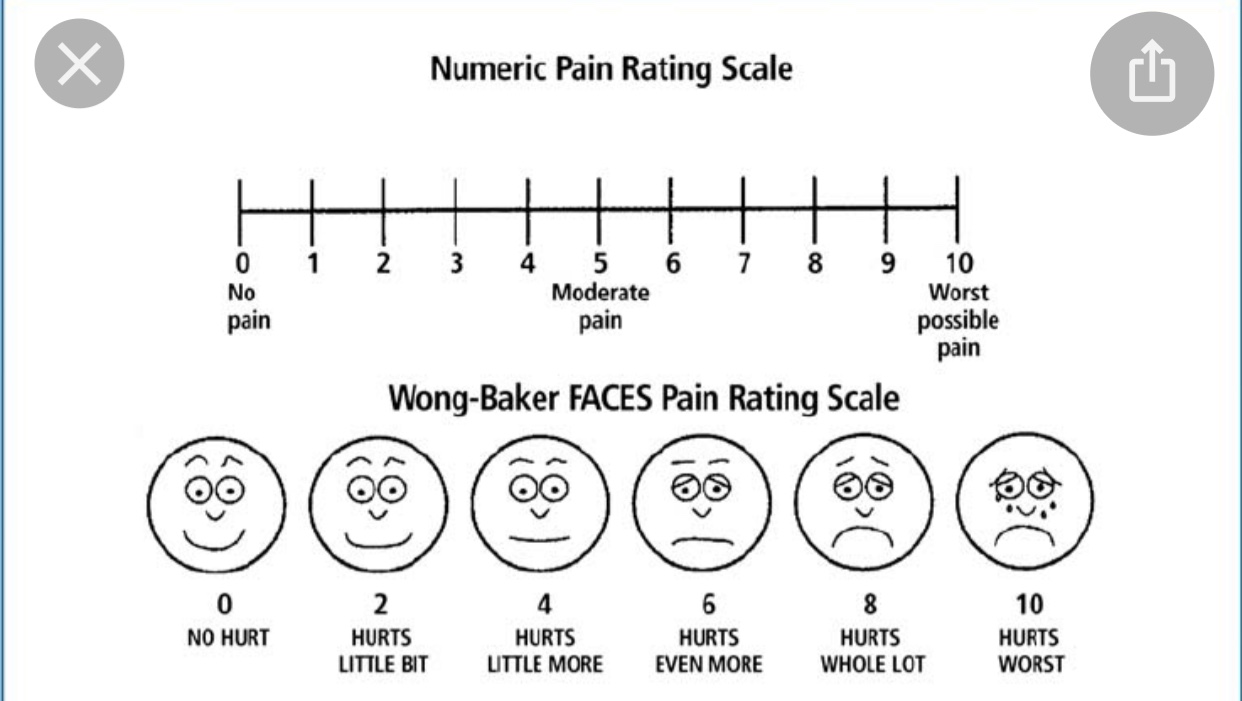** | | | | | | |

## Appendix IV: Immediately Post-intervention Questionnaire --Child/teenager

| 1. Under each heading, please tick **ONE** box that best describes your health **TODAY**. | |
| --- | --- |
| **Mobility (walking about)** | |
| I have no problem moving about. | 🞏 |
| I have some problems walking about. | 🞏 |
| I have a lot of problem walking about. | 🞏 |
| **Looking after myself** | |
| I have no problem washing or dressing myself. | 🞏 |
| I have some problem washing or dressing myself. | 🞏 |
| I have a lot of problems washing or dressing myself. | 🞏 |
| **Doing usual activities (for example, go to school, hobbies, sports, play, do things with family and friends)** | |
| I have no problem doing my usual activities. | 🞏 |
| I have some problem doing my usual activities. | 🞏 |
| I have a lot of problems doing my usual activities. | 🞏 |
| **Having pain or discomfort** | |
| I have no pain or discomfort. | 🞏 |
| I have some pain or discomfort. | 🞏 |
| I have a lot of pain or discomfort. | 🞏 |
| **Feeling worried, sad or unhappy** | |
| I am not worried, sad or unhappy. | 🞏 |
| I am a bit worried, sad or unhappy. | 🞏 |
| I am very worried, sad or unhappy. | 🞏 |
| We would like to know how good or bad your health is TODAY.  This line is numbered from 0 to 100.  100 means the best health you can imagine.  0 means the worst health you can imagine.  Please mark an X on the line that shows how good or bad your health is TODAY. |  |

2. Please tell us what you feel about wearing the Mollii suit at home in the past 4 weeks.

______________________________________________________________________________________________________________________________________________________

3. How would you rate the experience of wearing the Mollii suit in the last 4 weeks? 🞏Excellent 🞏Good 🞏Fair 🞏Poor

4. Was it difficult to get on/off the suit? 🞏Yes 🞏No

5. Was it difficult for your child to tolerate the suit? 🞏Yes 🞏No

6. How did you respond with the suit on/afterwards?

🞏Excellent 🞏Good 🞏Fair 🞏Poor

Please elaborate: ___________________________________________________________________________Did the suit meet your expectation? 🞏Yes 🞏No

1. How satisfied are you with the Mollii suit?

🞏Very satisfied 🞏Mostly satisfied 🞏Neutral/ mild dissatisfied 🞏Quite dissatisfied

1. Has the suit helped you?

| 🞏Yes, it helped a great deal |
| --- |
| 🞏Yes, it helped |
| 🞏No, didn’t really help |
| 🞏No, it seemed to make things worse |

9. Will you be keen to wear the Mollii suit again? 🞏Yes 🞏No

## Annex V: 1 month Post intervention Questionnaire--Child/teenager

| 1. Under each heading, please tick **ONE** box that best describes your health **TODAY**. | |
| --- | --- |
| **Mobility (walking about)** | |
| I have no problem moving about. | 🞏 |
| I have some problems walking about. | 🞏 |
| I have a lot of problem walking about. | 🞏 |
| **Looking after myself** | |
| I have no problem washing or dressing myself. | 🞏 |
| I have some problem washing or dressing myself. | 🞏 |
| I have a lot of problems washing or dressing myself. | 🞏 |
| **Doing usual activities (for example, go to school, hobbies, sports, play, do things with family and friends)** | |
| I have no problem doing my usual activities. | 🞏 |
| I have some problem doing my usual activities. | 🞏 |
| I have a lot of problems doing my usual activities. | 🞏 |
| **Having pain or discomfort** | |
| I have no pain or discomfort. | 🞏 |
| I have some pain or discomfort. | 🞏 |
| I have a lot of pain or discomfort. | 🞏 |
| **Feeling worried, sad or unhappy** | |
| I am not worried, sad or unhappy. | 🞏 |
| I am a bit worried, sad or unhappy. | 🞏 |
| I am very worried, sad or unhappy. | 🞏 |
| We would like to know how good or bad your health is TODAY.  This line is numbered from 0 to 100.  100 means the best health you can imagine.  0 means the worst health you can imagine.  Please mark an X on the line that shows how good or bad your health is TODAY. |  |

2. Will you be keen to wear the Mollii suit again? 🞏Yes 🞏No

3. Other comments:

_________________________________________________________________________________________________________________________________________________________________________________________________________________________________

## Appendix VI: Immediately Post intervention Questionnaire – PARENT

| 1. ICF | 0: No problem/ difficulty  1: Mild problem/ difficulty (present <25% time)  2: Moderate problem/difficulty (present 25-50% time)  3: Severe problem/ difficulty (present >50% time)  4: Complete problem/ difficulty (present >95% time) | | | | | |
| --- | --- | --- | --- | --- | --- | --- |
| **BODY FUNCTIONS** | | **0** | **1** | **2** | **3** | **4** |
| Does your child sleep enough? | | 🞏 | 🞏 | 🞏 | 🞏 | 🞏 |
| Does your child have problem falling asleep? | | 🞏 | 🞏 | 🞏 | 🞏 | 🞏 |
| Does your child have problem staying asleep? | | 🞏 | 🞏 | 🞏 | 🞏 | 🞏 |
| Does your child have good quality of sleep/ feel rested? | | 🞏 | 🞏 | 🞏 | 🞏 | 🞏 |
| Does your child have generalised pain? | | 🞏 | 🞏 | 🞏 | 🞏 | 🞏 |
| Does your child have problem with his/her tone of all muscles of body | | 🞏 | 🞏 | 🞏 | 🞏 | 🞏 |
| Does your child have difficulty with involuntary contractions of muscles? | | 🞏 | 🞏 | 🞏 | 🞏 | 🞏 |
| **ACTIVITIES & PARTICIPATION** | | **0** | **1** | **2** | **3** | **4** |
| Does your child have difficulty maintaining a sitting position (supported/ independent)? | | 🞏 | 🞏 | 🞏 | 🞏 | 🞏 |
| Is it easy to transfer your child? | | 🞏 | 🞏 | 🞏 | 🞏 | 🞏 |
| Is it easy to perform toileting needs of your child? | | 🞏 | 🞏 | 🞏 | 🞏 | 🞏 |
| Is there difficulty/ problem for your child to make basic interpersonal interactions? | | 🞏 | 🞏 | 🞏 | 🞏 | 🞏 |
| Is there difficulty/ problem for your child to make family relationships | | 🞏 | 🞏 | 🞏 | 🞏 | 🞏 |

| 2.Under each heading, please tick **ONE** box that best describes your child’s health **TODAY**. | |
| --- | --- |
| **Mobility (moving about)** | |
| My child has no problem moving about. | 🞏 |
| My child has some problems moving about. | 🞏 |
| My child has a lot of problem moving about. | 🞏 |
| **Looking after self** | |
| My child has no problem washing or dressing him/herself. | 🞏 |
| My child has some problem washing or dressing him/herself. | 🞏 |
| My child has a lot of problems washing or dressing him/herself. | 🞏 |
| **Doing usual activities (for example, go to school, hobbies, sports, play, do things with family and friends)** | |
| My child has no problem doing my usual activities. | 🞏 |
| My child has some problem doing my usual activities. | 🞏 |
| My child has a lot of problems doing my usual activities. | 🞏 |
| **Having pain or discomfort** | |
| My child has no pain or discomfort. | 🞏 |
| My child has some pain or discomfort. | 🞏 |
| My child has a lot of pain or discomfort. | 🞏 |
| **Feeling worried, sad or unhappy** | |
| My child is not worried, sad or unhappy. | 🞏 |
| My child is a bit worried, sad or unhappy. | 🞏 |
| My child is very worried, sad or unhappy. | 🞏 |
| We would like to know how good or bad your child’s health is TODAY.  This line is numbered from 0 to 100.  100 means the best health you can imagine.  0 means the worst health you can imagine.  Please mark an **X** on the line that shows how good or bad your child’s health is **TODAY**. |  |

3. What was a typical day like for you when you were involved in the Mollii suit study and your child wore the suit every day for one hour?

_________________________________________________________________________________________________________________________________________________________________________________________________________________________________

4. How would you rate the experience of wearing the Mollii suit in the last 4 weeks? 🞏Excellent 🞏Good 🞏Fair 🞏Poor

5. Was it difficult to get on/off the suit? 🞏Yes 🞏No

6. Was it difficult for your child to tolerate the suit? 🞏Yes 🞏No

7. How did your child respond with the suit on/afterwards?

🞏Excellent 🞏Good 🞏Fair 🞏Poor

Please elaborate: ______________________________________________________________________________________________________________________________________________________

8. Did the suit meet your expectation? 🞏Yes 🞏No

9. How satisfied are you with the Mollii suit?

🞏Very satisfied 🞏Mostly satisfied 🞏Neutral/ mild dissatisfied 🞏Quite dissatisfied

10. Has the suit helped your child?

| 🞏Yes, it helped a great deal |
| --- |
| 🞏Yes, it helped |
| 🞏No, didn’t really help |
| 🞏No, it seemed to make things worse |

11. Will you recommend the suit to another family? 🞏Yes 🞏No 12. Will you be keen for your child to wear the suit again? 🞏Yes 🞏No

13. Were there any other expected or unexpected reactions, difficulties, benefits? 🞏Yes 🞏No

______________________________________________________________________________________________________________________________________________________

14. Did you notice any changes in your child’s pain, mood, capabilities or any other aspect of their daily life across the four weeks? Please describe in detail.

_________________________________________________________________________________________________________________________________________________________________________________________________________________________________

15. What advice would you give to another family about the Mollii suit and ease of use, benefits or drawbacks on use?

_________________________________________________________________________________________________________________________________________________________________________________________________________________________________

16. What advice would you give to service providers/health professionals about the Mollii suit?

_________________________________________________________________________________________________________________________________________________________________________________________________________________________________

17. Please tell us how much you think you will pay for the rental of the Mollii Suit over 4 weeks.

___________________________________________________________________________

## Annex VII: 1month Post intervention Questionnaire – PARENT

| 1. ICF | 0: No problem/ difficulty  1: Mild problem/ difficulty (present <25% time)  2: Moderate problem/difficulty (present 25-50% time)  3: Severe problem/ difficulty (present >50% time)  4: Complete problem/ difficulty (present >95% time) | | | | | |
| --- | --- | --- | --- | --- | --- | --- |
| **BODY FUNCTIONS** | | **0** | **1** | **2** | **3** | **4** |
| Does your child sleep enough? | | 🞏 | 🞏 | 🞏 | 🞏 | 🞏 |
| Does your child have problem falling asleep? | | 🞏 | 🞏 | 🞏 | 🞏 | 🞏 |
| Does your child have problem staying asleep? | | 🞏 | 🞏 | 🞏 | 🞏 | 🞏 |
| Does your child have good quality of sleep/ feel rested? | | 🞏 | 🞏 | 🞏 | 🞏 | 🞏 |
| Does your child have generalised pain? | | 🞏 | 🞏 | 🞏 | 🞏 | 🞏 |
| Does your child have problem with his/her tone of all muscles of body | | 🞏 | 🞏 | 🞏 | 🞏 | 🞏 |
| Does your child have difficulty with involuntary contractions of muscles? | | 🞏 | 🞏 | 🞏 | 🞏 | 🞏 |
| **ACTIVITIES & PARTICIPATION** | | **0** | **1** | **2** | **3** | **4** |
| Does your child have difficulty maintaining a sitting position (supported/ independent)? | | 🞏 | 🞏 | 🞏 | 🞏 | 🞏 |
| Is it easy to transfer your child? | | 🞏 | 🞏 | 🞏 | 🞏 | 🞏 |
| Is it easy to perform toileting needs of your child? | | 🞏 | 🞏 | 🞏 | 🞏 | 🞏 |
| Is there difficulty/ problem for your child to make basic interpersonal interactions? | | 🞏 | 🞏 | 🞏 | 🞏 | 🞏 |
| Is there difficulty/ problem for your child to make family relationships | | 🞏 | 🞏 | 🞏 | 🞏 | 🞏 |

| 2.Under each heading, please tick **ONE** box that best describes your child’s health **TODAY**. | |
| --- | --- |
| **Mobility (moving about)** | |
| My child has no problem moving about. | 🞏 |
| My child has some problems moving about. | 🞏 |
| My child has a lot of problem moving about. | 🞏 |
| **Looking after self** | |
| My child has no problem washing or dressing him/herself. | 🞏 |
| My child has some problem washing or dressing him/herself. | 🞏 |
| My child has a lot of problems washing or dressing him/herself. | 🞏 |
| **Doing usual activities (for example, go to school, hobbies, sports, play, do things with family and friends)** | |
| My child has no problem doing my usual activities. | 🞏 |
| My child has some problem doing my usual activities. | 🞏 |
| My child has a lot of problems doing my usual activities. | 🞏 |
| **Having pain or discomfort** | |
| My child has no pain or discomfort. | 🞏 |
| My child has some pain or discomfort. | 🞏 |
| My child has a lot of pain or discomfort. | 🞏 |
| **Feeling worried, sad or unhappy** | |
| My child is not worried, sad or unhappy. | 🞏 |
| My child is a bit worried, sad or unhappy. | 🞏 |
| My child is very worried, sad or unhappy. | 🞏 |
| We would like to know how good or bad your child’s health is TODAY.  This line is numbered from 0 to 100.  100 means the best health you can imagine.  0 means the worst health you can imagine.  Please mark an **X** on the line that shows how good or bad your child’s health is **TODAY**. |  |

3. What advice would you give to service providers/health professionals about the Mollii suit?

_________________________________________________________________________________________________________________________________________________________________________________________________________________________________

4. Will you be keen for your child to wear the Mollii suit again? 🞏Yes 🞏No

5. Please tell us how much you think you will pay for the rental of the Mollii Suit over 4 weeks.

## ________________________________________________________________________________________________________________________________________________________________________________________________

Annex VIII

***Table 1 to show GMFM scores***

****Shaded cell indicates an improvement in score from baseline***

| Subject | Age range (years) | GMFCS | CP | GMFM Pre (Domain, %) | | | | | | GMFM post (Domain, %) | | | | | | GMFM 1-month post (Domain, %) | | | | | |
| --- | --- | --- | --- | --- | --- | --- | --- | --- | --- | --- | --- | --- | --- | --- | --- | --- | --- | --- | --- | --- | --- |
|  |  |  |  | A | B | C | D | E | Total score | A | B | C | D | E | Total score | A | B | C | D | E | Total score |
| 1 | 7-16 | III | Diplegia | 96.08 | 96.67 | 78.57 | 38.46 | 18.06 | 65.57 | 100.00 | 100.00 | 92.85 | 33.33 | 18.06 | 68.5 | 100.00 | 100.00 | 85.71 | 23.08 | 15.28 | 64.81 |
| 2 | 2-6 | III | Diplegia | 100.00 | 96.67 | 85.71 | 69.23 | 30.55 | 76.43 | 100.00 | 98.33 | 88.10 | 51.28 | 37.50 | 75.04 | 100.00 | 96.67 | 76.19 | 23.08 | 40.28 | 67.24 |
| 3 | 7-16 | III | Diplegia | 100.00 | 98.33 | 92.24 | 41.03 | 38.89 | 74.79 | $100.00$ | 96.67 | 85.71 | 41.03 | 22.22 | 69.13 | 100.00 | 100.00 | 78.57 | 30.77 | 20.83 | 66.03 |
| 4 | 7-16 | II | Diplegia | 100.00 | 95.00 | 90.48 | 64.01 | 48.61 | 79.64 | 96.08 | 90.00 | 95.24 | 56.41 | 58.33 | 79.32 | 100.00 | 100.00 | 100.00 | 74.36 | 56.94 | 86.26 |
| 5 | 2-6 | I | Right hemiplegia | 100.00 | 100.00 | 100.00 | 97.44 | 92.22 | 98.93 | 100.00 | 100.00 | 100.00 | 97.44 | 94.44 | 98.30 | 100.00 | 100.00 | 100.00 | 94.87 | 95.83 | 98.14 |
| 6 | 7-16 | II | Right hemiplegia | 100.00 | 100.00 | 100.00 | 100.00 | 88.89 | 97.78 | 100.00 | 100.00 | 100.00 | 100.0 | 94.44 | 98.89 | 100.00 | 100.00 | 100.00 | 100.00 | 94.44 | 98.88 |
| 7 | 7-16 | II | Diplegia | 100.00 | 100.00 | 90.48 | 79.49 | 61.11 | 86.21 | 100.00 | 100.00 | 100.00 | 84.62 | 58.33 | 88.59 | 100.00 | 100.00 | 97.62 | 69.23 | 58.33 | 85.04 |
| 8 | 7-16 | III | Triplegia | 100.00 | 92.16 | 78.57 | 61.53 | 25.00 | 72.69 | 100.00 | 100.00 | 92.86 | 53.84 | 27.78 | 74.90 | 100.00 | 95.00 | 83.33 | 58.97 | 33.33 | 71.12 |
| 9 | 7-16 | I | Diplegia | 100.00 | 100.00 | 100.00 | 89.74 | 88.89 | 95.73 | 100.00 | 100.00 | 100.00 | 94.87 | 94.44 | 97.86 | 100.00 | 100.00 | 100.00 | 94.8 | 83.33 | 71.13 |
| 10 | 7-16 | III | Diplegia | 92.16 | 91.67 | 66.68 | 2.56 | 9.72 | 52.56 | 100.00 | 91.67 | 69.05 | 5.13 | 8.33 | 54.84 | 94.12 | 95.00 | 26.19 | 17.95 | 13.89 | 49.43 |
| 11 | 7-16 | II | Diplegia | 100.00 | 96.67 | 90.48 | 82.05 | 43.05 | 82.45 | 100.00 | 100.00 | 95.24 | 62.10 | 52.78 | 82.42 | 100.00 | 95.00 | 88.10 | 69.23 | 51.39 | 80.74 |
| 12 | 7-16 | II | Diplegia | 100.00 | 100.00 | 100.00 | 89.74 | 65.28 | 91.00 | 100.00 | 100.00 | 100.00 | 94.87 | 66.67 | 93.40 | 100.00 | 100.00 | 100.00 | 87.18 | 61.11 | 89.66 |
| 13 | 7-16 | I | Monoplegia | 100.00 | 100.00 | 100.00 | 97.44 | 95.84 | 98.66 | 100.00 | 100.00 | 100.00 | 100.00 | 97.22 | 99.44 | 100.00 | 100.00 | 100.00 | 100.00 | 97.22 | 99.44 |
| 14 | 2-6 | II | Diplegia | 100.00 | 100.00 | 100.00 | 79.49 | 70.83 | 90.64 | 100.00 | 100.00 | 100.00 | 84.62 | 69.44 | 90.81 | 100.00 | 100.00 | 100.00 | 82.05 | 81.94 | 92.80 |
| 15 | 7-16 | II | Diplegia | 100.00 | 100.00 | 85.71 | 64.10 | 54.17 | 80.80 | 100.00 | 100.00 | 88.10 | 53.85 | 56.94 | 79.78 | 100.00 | 100.00 | 90.48 | 61.54 | 44.44 | 79.29 |
| 16 | 7-16 | II | Diplegia | 96.07 | 96.67 | 64.29 | 58.97 | 51.39 | 73.48 | 96.08 | 96.67 | 69.05 | 56.41 | 50.00 | 73.64 | 96.08 | 98.33 | 80.95 | 71.80 | 48.62 | 79.15 |
| 17 | 7-16 | II | Diplegia | 100.00 | 100.00 | 90.48 | 69.23 | 69.44 | 85.83 | 100.00 | 100.00 | 90.48 | 66.67 | 81.94 | 87.81 | 100.00 | 100.00 | 95.24 | 82.05 | 76.39 | 90.74 |
| 18 | 2-6 | III | Diplegia | 98.04 | 100.00 | 90.48 | 38.46 | 25.00 | 70.40 | 96.08 | 98.33 | 90.48 | 61.54 | 19.44 | 73.17 | 100.00 | 100.00 | 88.10 | 38.46 | 20.83 | 69.48 |
| 19 | 7-16 | II | Diplegia | 100.00 | 100.00 | 95.24 | 74.36 | 90.28 | 91.97 | 0 | 0 | 0 | 0 | 0 | 0 | 0 | 0 | 0 | 0 | 0 | 0 |
| 20 | 2-6 | II | Diplegia | 92.16 | 90.00 | 73.81 | 56.41 | 23.72 | 69.42 | 100.00 | 98.33 | 85.71 | 64.10 | 40.28 | 77.68 | 96.08 | 100.00 | 88.10 | 48.72 | 40.28 | 74.63 |

***Table 2 to show EQ5D-Y scores (Child)***

*Highlighted cell indicates an improvement in score

| No | Age range (years) | GMFCS | CP | Pre | | | | | | Post | | | | | | 1-month post | | | | | |
| --- | --- | --- | --- | --- | --- | --- | --- | --- | --- | --- | --- | --- | --- | --- | --- | --- | --- | --- | --- | --- | --- |
|  |  |  |  | Mobility | Looking After | Usual Activities | Having Pain | Feeling Worried/Sad | Total score | Mobility | Looking after | Usual Activities | Having Pain | Feeling Worried/Sad | Total score | Mobility | Looking After | Usual Activities | Having Pain | Feeling worried / Sad | Total score |
| 1 | 7-16 | III | Diplegia | 3 | 3 | 2 | 1 | 1 | 10 | 2 | 2 | 1 | 2 | 2 | 9 | 2 | 2 | 1 | 1 | 2 | 8 |
| 2 | 2-6 | III | Diplegia |  |  |  |  |  | 0 |  |  |  |  |  |  |  |  |  |  |  |  |
| 3 | 7-16 | III | Diplegia | 2 | 2 | 2 | 2 | 2 | 10 | 1 | 2 | 2 | 1 | 1 | 7 | 1 | 1 | 1 | 1 | 1 | 5 |
| 4 | 7-16 | II | Diplegia | 1 | 2 | 1 | 1 | 1 | 6 | 2 | 3 | 1 | 1 | 1 | 8 | 1 | 1 | 1 | 1 | 1 | 5 |
| 5 | 2-6 | I | Right hemiplegia | 1 | 1 | 1 | 1 | 1 | 5 | 1 | 1 | 1 | 1 | 1 | 5 | 1 | 1 | 1 | 2 | 1 | 6 |
| 6 | 7-16 | II | Right hemiplegia | 2 | 1 | 1 | 1 | 1 | 6 | 1 | 1 | 1 | 1 | 1 | 5 | 1 | 1 | 1 | 1 | 1 | 5 |
| 7 | 7-16 | II | Diplegia | 2 | 1 | 2 | 1 | 1 | 7 | 1 | 2 | 1 | 1 | 1 | 6 | 2 | 2 | 2 | 1 | 2 | 9 |
| 8 | 7-16 | III | Triplegia | 1 | 2 | 2 | 1 | 1 | 7 | 2 | 2 | 1 | 1 | 1 | 7 | 2 | 2 | 2 | 1 | 1 | 8 |
| 9 | 7-16 | I | Diplegia | 2 | 3 | 1 | 2 | 2 | 10 | 1 | 1 | 1 | 2 | 1 | 6 | 1 | 1 | 1 | 2 | 1 | 6 |
| 10 | 7-16 | III | Diplegia |  |  |  |  |  | 0 |  |  |  |  |  |  |  |  |  |  |  |  |
| 11 | 7-16 | II | Diplegia |  |  |  |  |  | 0 |  |  |  |  |  |  |  |  |  |  |  |  |
| 12 | 7-16 | II | Diplegia | 2 | 1 | 2 | 1 | 2 | 8 | 2 | 1 | 2 | 1 | 2 | 8 | 2 | 1 | 2 | 1 | 2 | 8 |
| 13 | 7-16 | I | Monoplegia | 2 | 1 | 1 | 2 | 1 | 7 | 1 | 1 | 1 | 1 | 1 | 5 | 1 | 1 | 1 | 1 | 1 | 5 |
| 14 | 2-6 | II | Diplegia |  |  |  |  |  | 0 | 2 | 1 | 2 | 2 | 2 | 9 | 1 | 1 | 1 | 1 | 1 | 5 |
| 15 | 7-16 | II | Diplegia |  |  |  |  |  | 0 |  |  |  |  |  |  |  |  |  |  |  |  |
| 16 | 7-16 | II | Diplegia | 1 | 1 | 2 | 1 | 1 | 6 | 1 | 1 | 1 | 1 | 1 | 5 | 1 | 1 | 1 | 2 | 2 | 7 |
| 17 | 7-16 | II | Diplegia | 2 | 1 | 1 | 2 | 2 | 8 | 1 | 1 | 1 | 2 | 2 | 7 | 2 | 1 | 1 | 1 | 1 | 7 |
| 18 | 2-6 | III | Diplegia | 1 | 1 | 1 | 1 | 1 | 5 | 1 | 1 | 1 | 2 | 1 | 6 | 2 | 2 | 2 | 1 | 1 | 8 |
| 19 | 7-16 | II | Diplegia |  |  |  |  |  |  | 1 | 2 | 2 | 1 | 1 | 7 |  |  |  |  |  |  |
| 20 | 2-6 | II | Diplegia |  |  |  |  |  |  |  |  |  |  |  |  |  |  |  |  |  |  |

***Table 3 to show EQ5D-Y scores (Parent)***

*Highlighted cell indicates an improvement in score

| Subject | Age range (years) | GMFCS | CP | Pre | | | | | | Post | | | | | | 1-month post | | | | | |
| --- | --- | --- | --- | --- | --- | --- | --- | --- | --- | --- | --- | --- | --- | --- | --- | --- | --- | --- | --- | --- | --- |
|  |  |  |  | Mobility | Looking After | Usual Activities | Having Pain | Feeling Worried/Sad | Total score | Mobility | Looking after | Usual Activities | Having Pain | Feeling Worried/Sad | Total score | Mobility | Looking After | Usual Activities | Having Pain | Feeling worried / Sad | Total score |
| 1 | 7-16 | III | Diplegia | 3 | 3 | 2 | 1 | 1 | 10 | 3 | 3 | 2 | 2 | 1 | 11 | 3 | 3 | 2 | 1 | 1 | 10 |
| 2 | 2-6 | III | Diplegia | 2 | 2 | 1 | 1 | 2 | 8 | 2 | 3 | 2 | 2 | 1 | 10 | 1 | 2 | 1 | 1 | 1 | 6 |
| 3 | 7-16 | III | Diplegia | 2 | 2 | 2 | 2 | 2 | 10 | 3 | 3 | 2 | 2 | 2 | 12 | 3 | 3 | 3 | 2 | 2 | 13 |
| 4 | 7-16 | II | Diplegia | 2 | 3 | 2 | 1 | 1 | 9 | 2 | 3 | 1 | 1 | 1 | 8 | 2 | 2 | 1 | 2 | 1 | 8 |
| 5 | 2-6 | I | Right hemiplegia | 1 | 2 | 2 | 1 | 1 | 7 | 1 | 2 | 1 | 1 | 1 | 6 | 1 | 1 | 1 | 1 | 1 | 5 |
| 6 | 7-16 | II | Right hemiplegia | 1 | 2 | 2 | 2 | 1 | 8 | 1 | 1 | 1 | 1 | 1 | 5 | 1 | 1 | 2 | 1 | 1 | 6 |
| 7 | 7-16 | II | Diplegia | 1 | 1 | 1 | 1 | 2 | 6 | 2 | 2 | 1 | 1 | 1 | 7 | 2 | 2 | 2 | 1 | 1 | 9 |
| 8 | 7-16 | III | Triplegia | 3 | 3 | 3 | 2 | 1 | 12 | 3 | 3 | 2 | 1 | 1 | 10 | 3 | 2 | 2 | 1 | 2 | 9 |
| 9 | 7-16 | I | Diplegia | 1 | 1 | 1 | 1 | 2 | 6 | 1 | 1 | 1 | 2 | 2 | 7 | 1 | 1 | 1 | 1 | 1 | 6 |
| 10 | 7-16 | III | Diplegia | 2 | 2 | 2 | 2 | 1 | 9 | 2 | 2 | 2 | 1 | 1 | 8 | 2 | 3 | 2 | 2 | 1 | 9 |
| 11 | 7-16 | II | Diplegia | 1 | 1 | 2 | 1 | 1 | 6 | 2 | 1 | 2 | 2 | 1 | 8 | 1 | 1 | 1 | 1 | 1 | 5 |
| 12 | 7-16 | II | Diplegia | 2 | 1 | 2 | 1 | 3 | 9 | 2 | 1 | 2 | 1 | 2 | 8 | 2 | 2 | 2 | 1 | 1 | 8 |
| 13 | 7-16 | I | Monoplegia | 1 | 1 | 1 | 1 | 1 | 5 | 1 | 1 | 1 | 1 | 1 | 5 | 1 | 1 | 1 | 1 | 1 | 5 |
| 14 | 2-6 | II | Diplegia | 1 | 2 | 2 | 1 | 1 | 7 | 1 | 2 | 2 | 1 | 1 | 7 | 1 | 2 | 2 | 1 | 1 | 7 |
| 15 | 7-16 | II | Diplegia | 2 | 2 | 2 | 2 | 2 | 10 | 2 | 2 | 1 | 1 | 3 | 9 | 2 | 2 | 1 | 1 | 2 | 8 |
| 16 | 7-16 | II | Diplegia | 1 | 1 | 1 | 1 | 1 | 5 | 1 | 1 | 1 | 1 | 1 | 5 | 1 | 1 | 1 | 2 | 2 | 7 |
| 17 | 7-16 | II | Diplegia | 1 | 1 | 1 | 2 | 2 | 7 | 2 | 2 | 2 | 2 | 2 | 10 | 2 | 2 | 2 | 2 | 2 | 10 |
| 18 | 2-6 | III | Diplegia | 2 | 2 | 2 | 2 | 1 | 9 | 2 | 2 | 2 | 2 | 1 | 9 | 2 | 2 | 2 | 1 | 1 | 8 |
| 19 | 7-16 | II | Diplegia | 2 | 2 | 2 | 2 | 2 | 10 | 2 | 2 | 2 | 1 | 2 | 9 |  |  |  |  |  | 0 |
| 20 | 2-6 | II | Diplegia | 2 | 2 | 1 | 1 | 2 | 8 | 2 | 1 | 2 | 1 | 1 | 7 | 2 | 1 | 1 | 1 | 1 | 7 |
